# Sex-differences in autonomic and cardiovascular responses to multimodal therapy in Parkinson’s disease: a pilot study

**DOI:** 10.1186/s12883-025-04281-7

**Published:** 2025-06-26

**Authors:** Franziska Siche-Pantel, Manfred Mühlenberg, Rüdiger Buschfort, Heinke Michels, Rasmus Jakobsmeyer, Julian Oesterschlink, Claus Reinsberger

**Affiliations:** 1 Department of Exercise and Health, Faculty of Science, Institute of Sports Medicine, Paderborn University, Warburger Str. 100, Paderborn, 33098 Germany; 2 Neurologic department, Gräfliche Kliniken, Marcus Klinik GmbH und Co. KG, Bad Driburg, 33014 Germany; 3 Neurologic department, Aatalklinik Wünnenberg GmbH, In Den Erlen 22, Bad Wünnenberg, 33181 Germany; 4 Dept. of Neurology, Division of Sports Neurology and Neurosciences, Mass General Brigham, Harvard Medical School, 55 Fruit Street, Boston, MA 02114 USA

**Keywords:** Parkinson’s disease, Sex differences, Cardiovascular dysregulation, Dysautonomia, Exercise

## Abstract

**Background:**

Parkinson’s Disease (PD) bears a variety of sex differences and is associated with cardiovascular dysregulation (CDR). Variation in the routinely assessed standard parameters heart rate (HR) and blood pressure (BP) seem not well understood within the frame of sex-specific developments under therapy. Parameters of heart rate variability (RMSSD) and electrodermal activity (meanEDA) may assist the understanding of underlying autonomic developments. This pilot study aims to describe sex-specific cardiovascular and autonomic responses to a multimodal inpatient rehabilitation program in patients with PD.

**Methods:**

Forty-one PD patients (24 male, 17 female) participated in a stationary, multimodal therapy intervention (MTI). Before and after MTI, HR, BP, RMSSD, and meanEDA were assessed in supine baseline (5 min of rest before orthostasis) and during supine adaption to rest (5 min of rest after orthostasis). Differences between baseline and adaption to rest as well as differences over time of MTI were calculated using Wilcoxon test; sex differences using Mann–Whitney-U test.

**Results:**

Before MTI, women’s supine HR (*p* = .034*; d = .17) and BP (*p* = .015*, d = 0.4) were significantly higher during adaption to rest than during baseline. After MTI, women’s supine HR (*p* = .020*; d = .84) and BP (*p* = .022*, d = 0.5) during adaption to rest had decreased significantly. Men’s HR and BP remained constant and without differences between the supine conditions. RMSSD and meanEDA remained steady in both sexes.

**Conclusion:**

The sex-specific responsiveness to MTI supports the concept of sex-sensitive therapeutic management for cardiovascular symptoms in PD. In both sexes, peripheral cardiovascular outcomes appeared not attributable to corresponding outcomes in autonomic regulation. Further examination of autonomic parameters could provide a foundation for developing therapeutic approaches that address central nervous system mechanisms.

The study was officially registered (08/2020). The data supporting the findings of this study are available under http://apps.who.int/trialsearch/ under trial number *DRKS00022773.*

**Supplementary Information:**

The online version contains supplementary material available at 10.1186/s12883-025-04281-7.

## Introduction

Parkinson’s Disease (PD) is considered one of the fastest-growing neurologic movement disorders worldwide [1], and bears a variety of sex differences. Men are predominantly affected [2], display larger preventive effects induced by physical activity [3], and demonstrate differently distributed motor and non-motor symptoms in comparison to women [4].

However, sex-specific responses to therapy remain less well understood, and few therapeutic recommendations differentiate between sexes. Recognizing this gap, multiple national and international organizations advocate for sex-sensitive medical research: the World Health Organization calls for increased inclusion of female patients in clinical studies [5]; the Parkinson’s Foundation (USA) has established an agenda to improve care and research for women with PD [6]; and scientists emphasize the need for sex-sensitive therapy management in PD [7, 8].

Cardiovascular dysregulation (CDR) is present in all synucleinopathies. In PD, CDR includes dysregulated heart rate (HR) and blood pressure (BP) [9, 10].

1. Already in the early stages of the disease [11], HR dysregulation is caused by impaired sympathetic and parasympathetic cardiac function [12], that may be attributed to various factors, such as disrupted pre- and postganglionic acetylcholine and norepinephrine release during sympathetic neurotransmission, or local and central impairments caused by Lewy bodies in autonomic structures [12]. Albeit the correlation may be weaker in women [13], a resulting symptom, such as high HR in resting states, is associated with an increased risk of mortality [14, 15] and can, therefore, be considered a crucial target in therapy.
2. Neurogenic blood pressure dysregulation presents as multiple hemodynamic symptoms with the associated risks of falls, trauma, increased mortality, and severe impact on the patient’s well-being [16–18]. Their pharmacological treatment targets opposing effects, which complicates clinical implementation [16, 17]; therefore, exercise therapy holds significant therapeutic value. Especially, hypertension in supine positions is considered a central parameter of CDR in PD [10] that appears to be based on impaired baroreflex function. However, the underlying pathophysiology remains insufficiently explored [19].

Even though the symptoms of CDR are increasingly well described, understanding CDR within specific autonomic subsystems remains challenging since cardiovascular regulation is considered a complex autonomic interaction [20] and routine assessments do not distinguish sufficiently between sympathetic and parasympathetic innervation. The regulation of autonomic functions occurs in an organ system- and task-specific manner [21, 22]. However, across different tasks, control appears to be mediated by a central autonomic network [23]. Analyzing peripheral parameters from autonomically regulated body systems might provide insights into a therapy-induced alteration of central autonomic control. Hence, in this study, two parameters with distinct autonomic innervation (sympathetic electrodermal activity and parasympathetic cardiac activity) were selected, alongside HR and BP, to provide a depiction of autonomic regulation in men and women under the influence of a therapy intervention. In this study, a certified, interdisciplinary, multimodal therapy intervention (MTI) was selected. Given the underrepresentation of women in clinical research and the documented sex differences in PD, we hypothesized that men and women would exhibit distinct cardiovascular responses to a standardized intervention (MTI), potentially driven by differences in autonomic control.

## Materials and methods

### Subjects

Patients diagnosed with idiopathic PD were recruited, and patients with atypical Parkinsonian Syndromes (e.g., Multiple System Atrophy, Lewy Body Dementia, Progressive Supranuclear Palsy, Corticobasal Ganglionic Degeneration, etc.) were excluded from the study. All diagnoses were given by the patient’s primary neurologist prior to the study. Patients with essential tremor, Parkinsonism, or further forms of PD (monogenetic, vascular, drug-induced, toxin-induced PD) were not being treated in the clinics at the time of the study and, therefore, were not part of the investigated patient group. None of the patients had been treated with invasive therapies (deep brain stimulation). Concurrent participation in other interventional studies was not allowed. All patients were asked to give written consent about their participation and the use of their data. If they were unable to understand the testing protocol and instructions (e.g., due to psychological conditions), they were excluded.

Given that a wide range of age-related and PD-related comorbidities is entirely typical within this patient population, these conditions were not used as exclusion criteria. 34% of the patients (42% of males, 24% of females) were previously diagnosed with arterial hypertension (Table 1). Further patient information containing comorbidities, medication intake, as well as previous participation in exercise therapy and informal physical activity, is available in the supplementary material of this manuscript.

**Table 1:** Demographic data; all included patients; mean values with standard deviations of age, duration of PD diagnosis and Body Mass Index, plus numbers of patients previously diagnosed with arterial hypertension and numbers of patients who received hypotensive agents (includes patients receiving antihypertensive medication for indications other than arterial hypertension, such as heart failure or coronary artery disease)

	*n*	Age [years]	Diagnosed since [years]	Body Mass Index [kg/m^2^]	Arterial Hypertension [*n*/%]	Hypotensive Agents [*n*/%]
total sample	41	70.3 ± 9.7	6.0 ± 6.6	27.1 ± 5.4	14/34	23/56
male	24	70.8 ± 10.3	5.9 ± 6.8	28.0 ± 5.5	10/42	15/63
female	17	69.9 ± 9.5	6.1 ± 6.6	25.9 ± 5.1	4/24	8/47

If the patients’ stated biological sex (including primary and secondary sexual characteristics) matched their gender identity and there were no known abnormalities in sex chromosomes, patients were conclusively allocated into one of two groups (male/female).

Since the primary focus of our research was not to evaluate the overall effectiveness of MTI but rather to investigate whether men and women with PD respond differently to it, a control group without treatment was not included.

### Experimental design

Between 08/2020 and 10/2021, all PD patients who underwent a certified, inpatient, multimodal, and interdisciplinary PD therapy (MTI) in one of two cooperating neurological rehabilitation centers in Germany were screened to be recruited. Pre-testing (before MTI) was performed within the first 48 h after hospital admission prior to the initialization of treatment. Post-testing (after MTI) was performed within 48 h before discharge from the inpatient stay. Both testing procedures followed the same protocol and occurred at a similar time of day with respect to medication intake, mealtime, caffeine consumption, and therapy. All patients completed the testing procedure in the on-stages of their PD medication.

### Multimodal therapy intervention (MTI)

MTI and testing procedures were performed in two rehabilitation centers that are certified to treat PD patients with an inpatient, multimodal, interdisciplinary approach. According to the official requirements of certified MTI in Germany, treatment was governed by a leading neurologist and contained at least three non-physician-led approaches. Physio- and ergotherapy were mandatory and had to be complemented by sports-, speech- or art therapy. If prescribed, patients also took part in psychosocial conversation therapy and consulting services (e.g., provision of aid). All therapy units were led by qualified personnel, and patients received a variety of evidence-based interventions like balance, strength, endurance, and gait training as well as walking classes, aqua aerobics, relaxation techniques, and stimulation of sensory perception. They were additionally allowed to use gyms and pools unsupervised in their spare time. Assignments to particular interventions as well as their duration and frequency (duration of stay: three to five weeks; training frequency: at least 7.5 h/week, thereof at least five hours/week individual therapy) were determined by the physician’s recommendation. The intensity and conduction of treatment were arranged by the therapeutic personnel.

PD symptoms could be pharmacologically treated under the physician’s care. Drug treatment contained the use of antihypertensive agents (in 63% of males, 47% of females. During our study, no adjustments of PD medication or antihypertensive agents were performed. A Bonferroni-Holm-corrected regression analysis did not reveal any significant associations between the use of antihypertensive medication and the study outcomes (Additional file 1). Therefore, it can be assumed that potential changes in study outcomes are primarily attributable to other therapeutic components, mainly physical activity. Yet, this study aims to describe the potentially sex-differentiated response to treatment rather than quantifying the direct associations between individual symptoms and specific treatment components.

### Testing procedures

Testing was performed by the study personnel. After giving consent to the procedure, patients strapped on a wireless wrist sensor (Empatica® E4). To reduce movement artifacts, the sensor was worn on the arm less affected by tremor. If no tremor occurred, the non-dominant wrist was chosen. The sensor was given a 30-min calibration time.

Patients were then asked to perform a sequence of postural conditions: 10 min of supine rest (baseline before orthostasis), 5 min of upright standing (orthostasis), and 5 min of supine lying (adaption to rest after orthostasis). Supine positions were performed with closed eyes, no speaking, and without cognitive tasks. The transfer between supine and upright positions was made swiftly, independently, and without interruptions.

Orthostasis was selected as a preceding stressor for the patient’s adaptation to rest, as it replicates a common everyday positional change that demands cardiovascular regulation due to significant blood volume redistribution within the body.

### Data analysis

BP was measured manually by stethoscopy on the upper arm (Riva-Rocci measurement). HR, RMSSD, and meanEDA were captured with the Empatica®E4 wrist sensor. The wearable is a class IIa Medical Device in the EU (CE Cert. No. 1876/MDD), designed for continuous use. In sitting rest positions, it shows high validity in heart rate variability and medium validity in electrodermal activity measurements [24]. In contrast to recording autonomic function via microneurography, the wearable operates in a non-invasive manner.

#### Blood pressure (BP)

BP was measured contralateral to the arm where the wrist sensor was worn. Only the systolic blood pressure was taken into statistical analysis, as it is a crucial marker in the diagnosis of neurogenic hypertension in distinction to essential hypertension [25], and is considered a greater risk factor in the elderly than the diastolic value [26]. In the supine baseline state before orthostasis, systolic pressure after two and ten minutes was averaged into one mean value to represent the baseline resting condition. Systolic BP higher than 140mmHg was categorized as hypertensive [26]. During adaption to supine rest after orthostasis, systolic pressure after each of five minutes was averaged into one mean value. A lowered BP after MTI was interpreted as improved rest/improved adaption to rest.

#### Heart rate (HR)

HR was taken from the blood volume pulse data sets measured by the wrist sensor at a sampling frequency of 64 Hz. The last five minutes of supine rest before orthostasis (baseline) as well as the first five minutes of supine rest after orthostasis (adaption to rest) were analyzed by generating an average HR for each of the supine conditions. A lowered HR after MTI was interpreted as improved rest/improved adaption to rest.

#### Root mean sum of squared distance (RMSSD)

RMSSD was chosen to depict parasympathetic cardiac activity, since time-domain assessments of heart rate variability in general [27] and especially short-term RMSSD recordings [28] are suitable to characterize cardiac dysregulation in PD. All postural conditions allowed spontaneous breathing, as RMSSD is considered robust regarding respiratory sinus arrhythmia [29] but debated for not properly assessing parasympathetic reactivity during deep breathing [30]. Via reflective photoplethysmography, the blood volume pulse was detected with a sampling frequency of 64 Hz. For cleaning the recorded data from artifacts, predefined settings for medium threshold-based correction in Kubios® (Kubios® HRV Software; Version: HRV Premium 3.5.0) were used on the inter-beat-intervals and additionally checked manually to ensure maximum data length within the permitted settings. Increased RMSSD after MTI was interpreted as improved rest/improved adaption to rest.

#### Electrodermal activity (meanEDA)

Since there are no purely sympathetically innervated cardiac parameters, tonic electrodermal activity was chosen to represent sympathetic autonomic activity [31]. With a 4 Hz sampling frequency, the dry silver-plated electrodes detected skin conductance in a range of 0.01 to 100 µS. The signal was cut into five-minute segments (last five minutes before orthostasis; first five minutes after orthostasis) and cleaned from artifacts using a coded script (Python Software Foundation; Version 3.9.12). A low-pass filter (Butterworth Filter; frequency 0.4 Hz; filter order 4) was applied to reduce noise. Visually detected artifacts were edited via interpolation. Lowered meanEDA after MTI was interpreted as improved rest/improved adaption to rest.

#### Data selection

The coded script allowed a simultaneous depiction of HR, RMSSD, and meanEDA. Only those data segments that were free of both artifacts in the blood volume pulse signal as well as in the EDA signal were taken into statistical calculation. A minimum of four minutes of clean data signal was required within each five-minute segment (last five minutes before orthostasis; first five minutes after orthostasis). Based on the resulting time-wise homogeneity of all data sets, no further time-related standardization was performed.

### Statistical analysis

Data was analysed with IBM® SPSS® Statistics software v.28 (IBM® Corporation, Armonk, New York, USA). Tables and figures were designed in MS Office.

After assessing normality using the Shapiro–Wilk and Kolmogorov–Smirnov tests, along with the Lilliefors test, differences between time points were analyzed using the Wilcoxon test. Sex differences within pre and post data sets were wielded with the Mann–Whitney U-test. To account for covariates (age, body mass index, duration of Parkinson’s disease, diagnosed cardiovascular comorbidities, and intake of antihypertensive medication), multiple linear regression analyses were performed. To adjust for multiple comparisons across outcome parameters, Bonferroni-Holm correction was applied post hoc. A representative example illustrating the association between the covariate'intake of antihypertensive medication'and the outcome parameters is provided in the additional files.

Missing data was caused by organisational instead of compliance-related issues, which justified an intention-to-treat analysis with a test-by-test exclusion to avoid data loss.

Due to the explorative character of the study, all calculations were carried out with two-tailed significance levels. In Mann–Whitney-U tests with more than 30 subjects, asymptotic significance was reported; in those with under 30 subjects, the exact significance is given. In all calculations, levels of significance were defined as *p* ≤ 0.05*. Cohen’s d was used to determine effect sizes (< 0.2: no effect; 0.2–0.5: small effect; 0.5–0.8: moderate effect; ≥ 0.8: strong effect).

## Results

### Demographic data

**Fig. 1:**
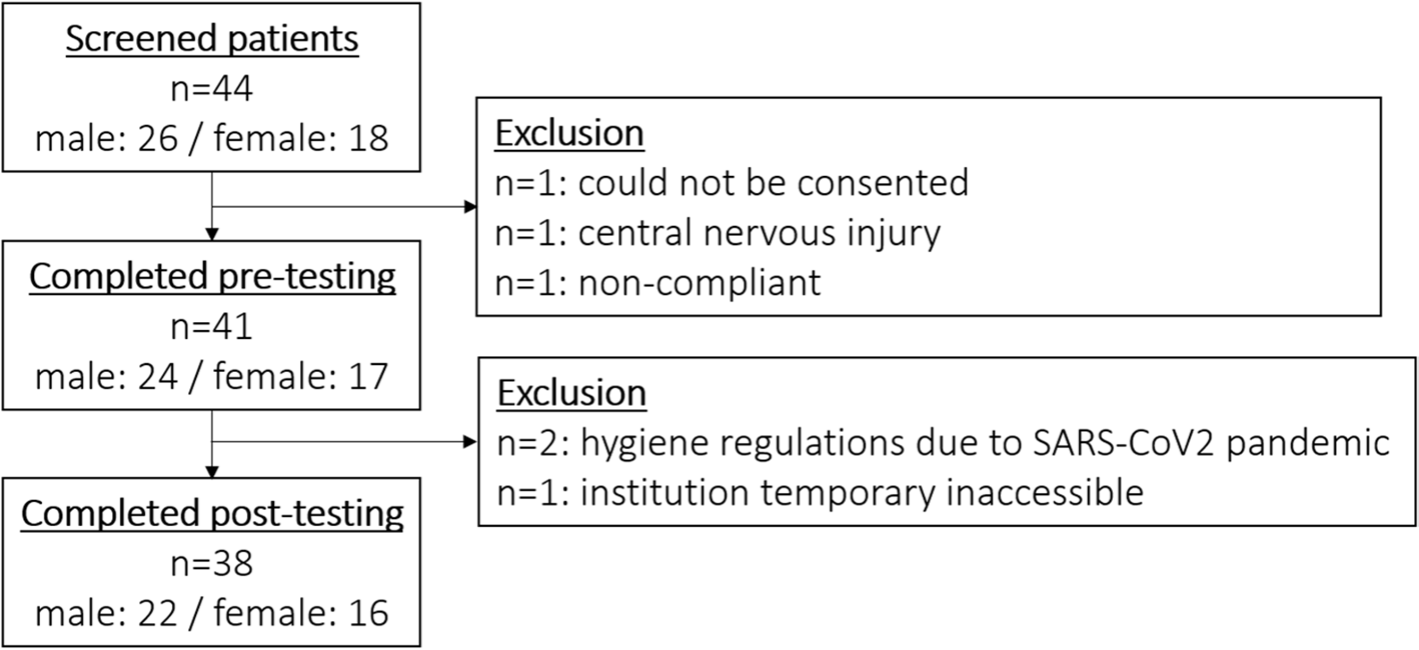
Participation and drop-out numbers; 44 patients were approached; three were excluded at pre-testing and three dropped out until post-testing; due to incomplete data sets, n-numbers in statistical calculations may deviate from this figure

Ultimately, 41 patients were enrolled (Table 1), and 38 completed post testing (Fig. 1). Due to occasional scheduling conflicts during busy therapy routines, some patients were unable to complete all assessments, resulting in incomplete data sets in pre as well as in post testing. Therefore, n-numbers in statistical calculations may deviate from Fig. 1.

### Blood pressure (BP)

In the baseline state of supine rest, neither group showed hypertensive systolic BP nor changes in BP after MTI (Additional file 2).

**Fig. 2:**
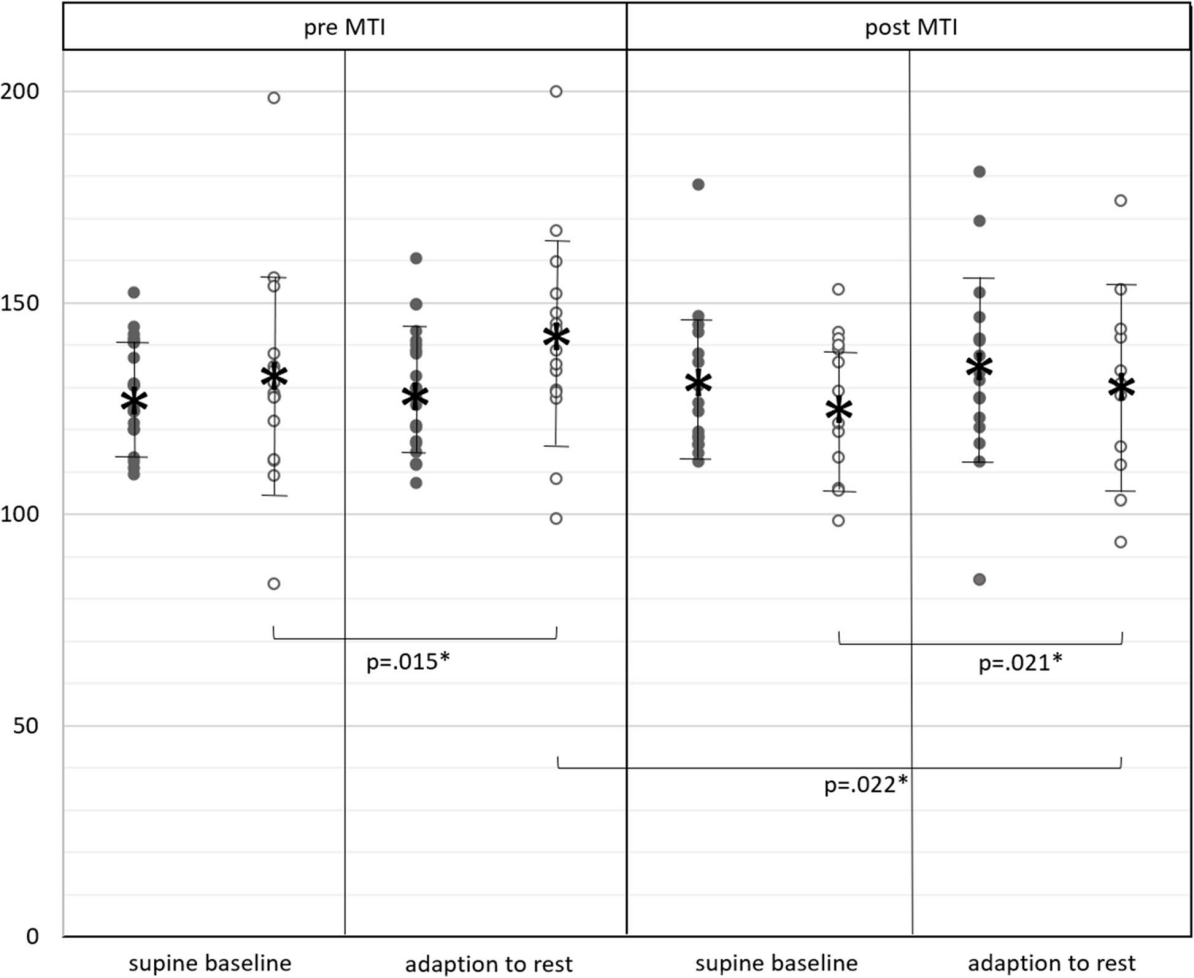
Systolic blood pressure; supine blood pressure in men (black dots) and women (white dots) in supine baseline state (before orthostasis) and during adaption to rest (after orthostasis), before (pre) and after (post) MTI; vertical bars with asterisk: mean ± standard deviation; horizontal brackets: significant differences

During adaption to rest, women presented with significantly higher systolic BP than in their baseline state (Fig. 2; Additional file 2). This was observed both in pre-testing (*p* = 0.015*, d = 0.4) as well as in post-testing (*p* = 0.021*, d = 0.3). From pre to post testing, women had significantly lowered their systolic BP during adaption to rest (*p* = 0.022*, d = 0.5). Men’s BP did not show differences over time of MTI or between baseline and adaption to rest. Supine BP during adaption to rest did not differ between men and women (pre-testing: *p* = 0.09, d = 0.64; post-testing: *p* = 0.753, d = −0.18).

### Heart rate (HR)

**Fig. 3:**
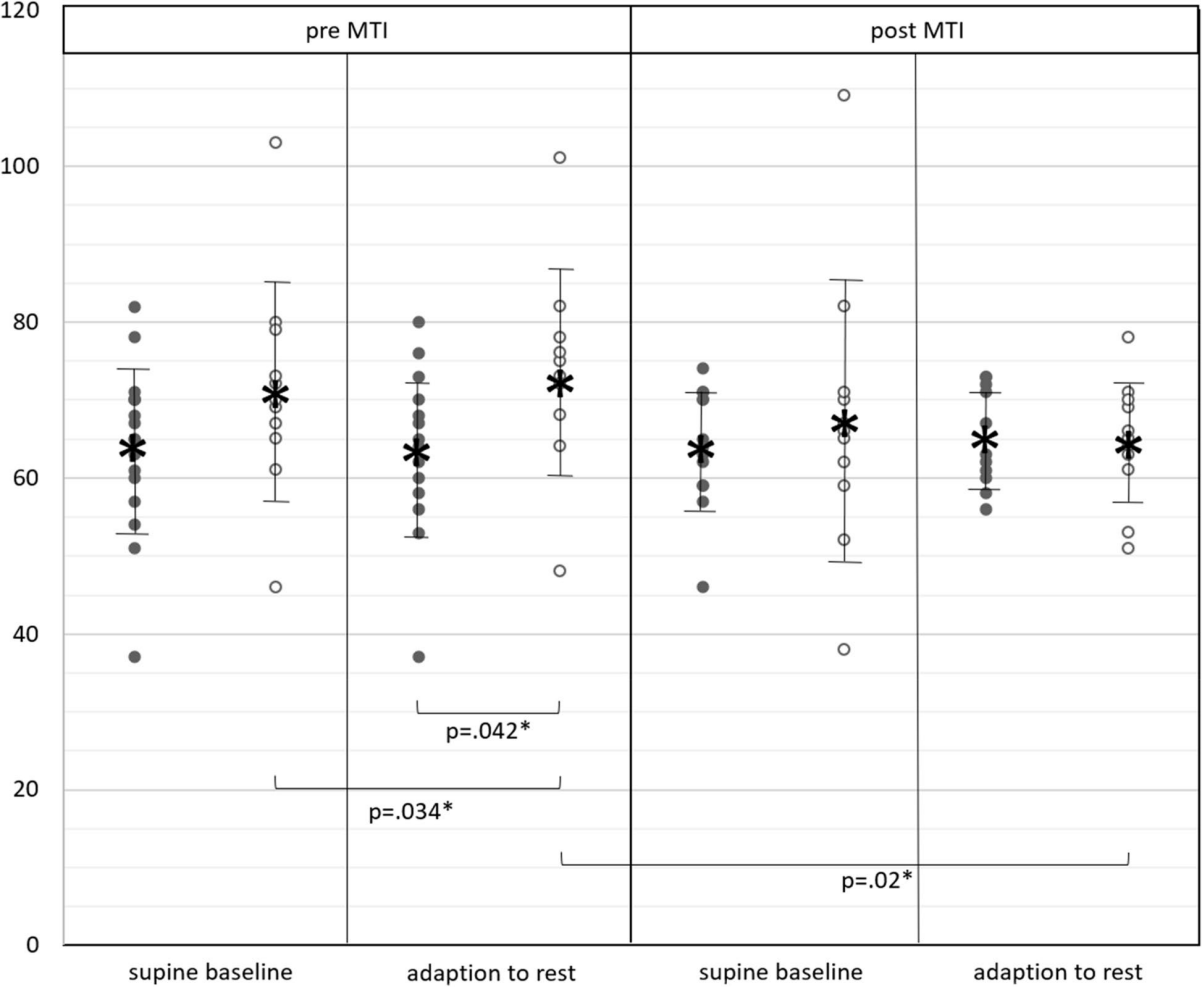
Heart rate; heart rate in men (black dots) and women (white dots) in supine baseline state (before orthostasis) and during adaption to rest (after orthostasis), before (pre) and after (post) MTI; vertical bars with asterisk: mean ± standard deviation; horizontal brackets: significant differences

In the baseline state of supine rest, neither significant changes over time of MTI nor significant differences between males and females were observed (Fig. 3; Additional file 2).

Before MTI, women’s HR during adaption to rest was significantly higher than in the baseline state (*p* = 0.034*; d = 0.17) (Fig. 3; Additional file 2) and significantly higher than in men (sex difference during adaption to rest: *p* = 0.042*, d = 0.85). This sex difference did not occur in post-testing (*p* = 0.949; d = −0.07), as women’s HR during adaption to rest had decreased significantly after MTI (*p* = 0.020*; d = 0.84).

Men’s HR did not show differences over time of MTI or between baseline and adaption to rest.

### Root mean sum of squared distance (RMSSD)

**Fig. 4:**
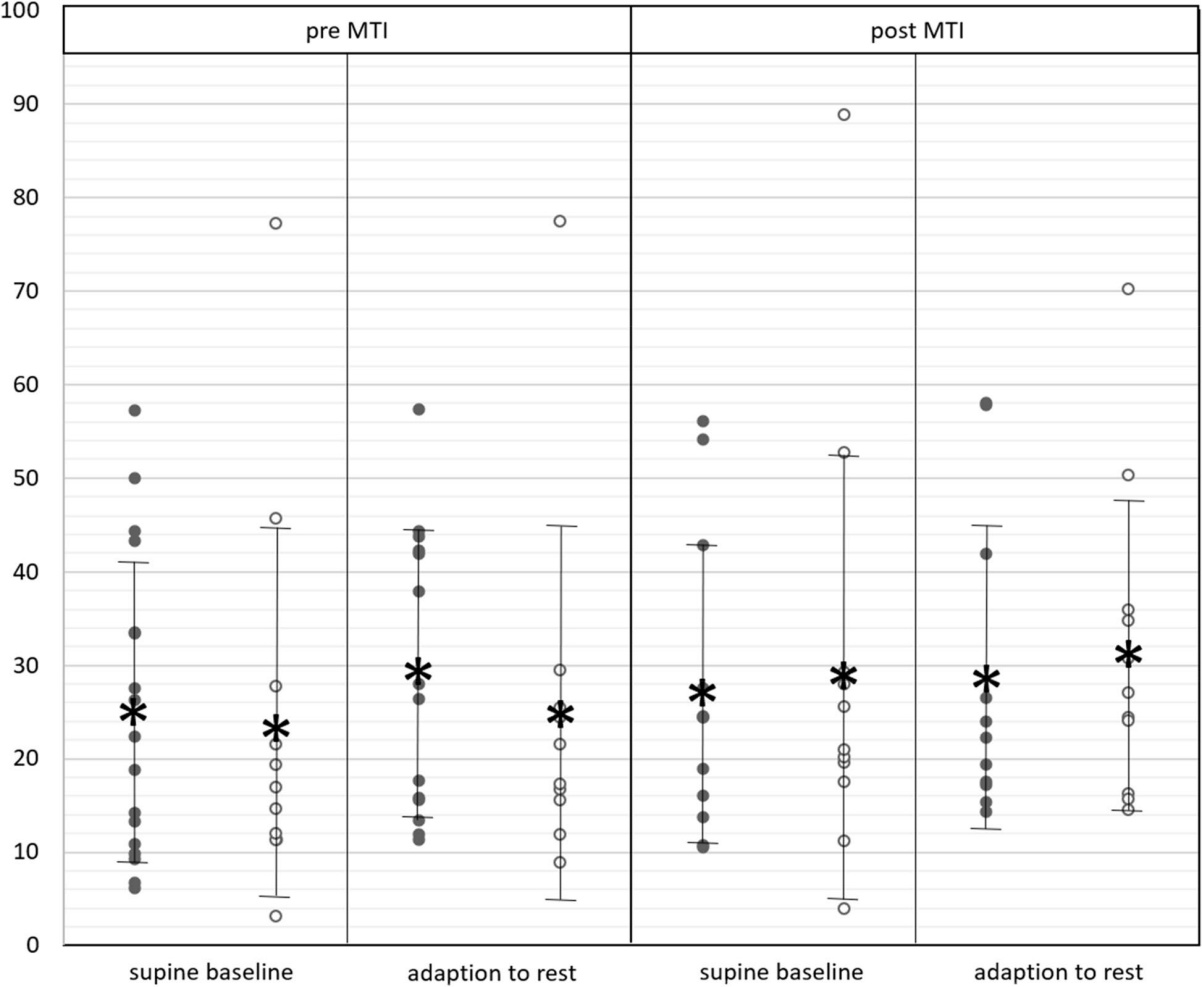
RMSSD; RMSSD in men (black dots) and women (white dots) in supine baseline state (before orthostasis) and during adaption to rest (after orthostasis), before (pre) and after (post) MTI; vertical bars with asterisk: mean ± standard deviation; horizontal brackets: significant differences

Neither in the baseline state nor during adaption to rest, did RMSSD values show differences over time of MTI or between men and women (Fig. 4; Additional file 2).

### Electrodermal activity (meanEDA)

**Fig. 5:**
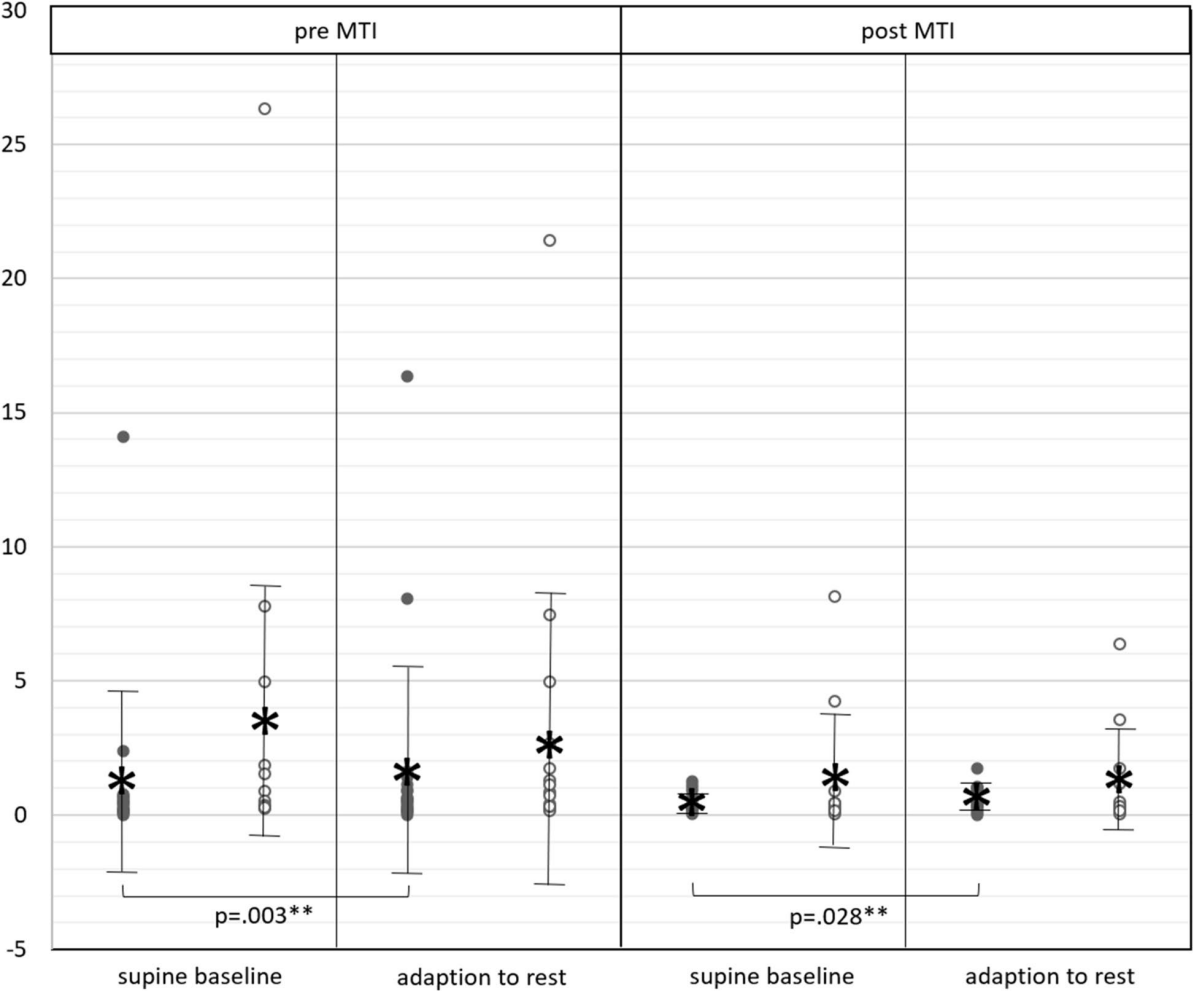
meanEDA*;* meanEDA in men (black dots) and women (white dots) in supine baseline state (before orthostasis) and during adaption to rest (after orthostasis), before (pre) and after (post) MTI; vertical bars with asterisk: mean ± standard deviation; horizontal brackets: significant differences

Neither in the baseline state nor during adaption to rest did meanEDA change over time of MTI or differ between men and women (Fig. 5; Additional file 2).

In men, meanEDA presented significantly higher during adaption to rest than in the supine baseline state; both before (*p* = 0.003**; d = 0.11) and after MTI (*p* = 0.028*; d = 0.22) (Fig. 5; Additional file 2).

### Correlations with covariates

No statistically significant associations with age, body mass index, disease duration, antihypertensive medication, or diagnosed cardiovascular comorbidities were observed for any of the outcome parameters in the linear regression analyses, after Bonferroni-Holm correction (Additional file 1).

## Discussion

### Cardiovascular and autonomic dysregulation in women

In women, HR and BP followed a similar pattern: (1) Before MTI, adaption to rest after orthostasis was accompanied by significantly higher supine BP and supine HR in comparison to the baseline states. These findings differed in comparison to males who did maintain their baseline values during adaption to rest. (2) Women’s BP and HR during adaption to rest then decreased significantly until after MTI. Even though women’s BP during adaption to rest remained higher than in the baseline state, it still fell below the threshold for hypertension in supine baseline. Therefore, MTI could be labeled effective in lowering women’s HR and BP during adaption to supine rest.

Improvements in HR and BP may indicate enhanced autonomic function, given that these parameters are modulated by both sympathetic and parasympathetic branches of the autonomic nervous system. However, no improvements in sympathetic (meanEDA) or parasympathetic (RMSSD) activation were found after MTI.

Even though dysregulation in the electrodermal system would not necessarily correlate with cardiovascular parameters [21], meanEDA was anticipated to depict sympathetic autonomic regulation in supine positions. The parameter’s sensitivity against uncontainable disturbances, like its reliance on central processes from the amygdala [32], lower skin conductance of aged skin [33], as well as high inter- and intraindividual variances may have prevented such observations.

A therapy-induced effect on parasympathetic cardiac regulation would have been confirmed by increased RMSSD in supine positions, which could be more suitable to predict outcomes like mortality than HR and BP alone [34]. Since women’s RMSSD values were comparable to those of the elderly without PD [35] and did not change over MTI, their improved BP and HR did not seem to be based on affected parasympathetic regulation either.

To intentionally provoke the improvement of cardiovascular dysfunction in women and to better understand potential mechanisms of central nervous control, a further understanding of the underlying mechanisms induced by therapy seems necessary. Future research might utilize a more time-sensitive measuring design that distinguishes between initial vagal and consecutive sympathetic regulation of HR [20] to better detect potential autonomic improvements. In addition, a sex-sensitive assessment of well-evaluated parameters like stress, heart volume, baroreflex sensitivity, and overall aerobic fitness may enable researchers to quantify the mechanisms affecting BP and HR in women [36] [17] [37]. This understanding is crucial for developing reproducible treatment outcomes and targeting individual patients who remain with pathologic HR and BP values after MTI (Additional file 2).

### Cardiovascular and autonomic dysregulation in men

In men, HR, BP, RMSSD, and meanEDA did not alter over time of MTI, indicating that no improvements in cardiovascular parameters or autonomic regulation were achieved. This might be due to a ceiling effect since men’s HR [14] and BP [26] did not approach critical marks at any point. Also, RMSSD values were equivalent to comparison data from the elderly without PD [35] and therefore suggested the absence of pathological significance, although group analyses may have concealed individual impairments. One potential reason for men’s consistent cardiovascular parameters might be their better representation in clinical research in comparison to women. It is highly possible that men were diagnosed earlier, got more specific care, and better-tailored treatment options prior to taking part in MTI [8].

During adaption to rest, men presented with higher meanEDA in comparison to their baseline states. This could imply impaired sympathetic regulation, which did not improve under MTI. However, meanEDA represents slower changes in sympathetic innervation [31]. Rising meanEDA could as well be a result of gradually accumulated sudor around the wrist sensor and therefore depict a physiologic reaction to prior upright standing. As there is no valid comparison data that describe standard electrodermal activity, it remained uncertain if neurogenic dysregulation during adaption to rest was present in men. If so, it did not show up in concurrently increased HR and BP and therefore did not allow conclusions about CDR.

## Conclusion

Men and women presented with distinct cardiovascular adaptions to supine rest and responded differently to MTI—highlighting the need for sex-sensitive therapeutic management of cardiovascular symptoms in PD. However, implementing sex-specific treatment strategies in clinical practice remains challenging without a deeper understanding of the underlying mechanisms. In both sexes, peripheral cardiovascular outcomes did not correspond to outcomes in parameters of autonomic regulation.

Given the neurodegenerative nature of PD, where restoring damaged autonomic structures may not be feasible through therapy, the ability to enhance heart rate and blood pressure without necessarily influencing central autonomic factors appears promising. However, considering that the absence of changes in RMSSD and meanEDA may also reflect methodological limitations, further research into autonomic control remains essential to elucidate cardiovascular regulation under therapy.

Building on pioneering approaches from epilepsy research [38], multimodal canonical correlation analyses could be employed to assess linear relationships between diverse autonomic parameters. A stronger correlation following MTI could indicate enhanced interaction between autonomic outcomes, reflecting improved integration within the central autonomic network. Such insights may contribute to the development of targeted therapeutic strategies that not only optimize peripheral cardiovascular function but also modulate central autonomic control in men and women with PD.

## Limitations

Our study is limited by the selection of patients. Data collection took place amidst the SARS-CoV2 pandemic in 2020/2021, with the possibility that more vulnerable patients and/or those who did not contemporarily rely on immediate care may have postponed their hospital stay to periods later on.

MTI is an officially certified intervention in Germany. The study results may therefore not be unconditionally transferable to other countries or healthcare systems.

Patients in our cohort were diagnosed with further age-related conditions and comorbidities that required drug treatment and may have affected study outcomes. This reflects a common characteristic of the PD patient population, which inherently limits the potential for stricter participant selection. However, we did not find any correlations between medication intake and study outcomes. Detailed information on the frequency of comorbidities and medications can be found in the supplementary material.

Since the primary focus of our research was not to evaluate the overall effectiveness of MTI but rather to investigate whether men and women with PD respond differently to a standardized, multimodal therapy approach, a control group without treatment was not included. Baseline measurements served as an internal control to assess treatment effects. Nonetheless, the lack of a control group, combined with the small overall cohort, particularly the limited number of female participants, limits the generalizability of our findings, and results should therefore be interpreted with caution. Future studies should be designed to overcome these limitations.

The study aimed to generate pilot results regarding the necessity of sex-sensitive treatment in PD patients. In future studies, a larger cohort could substantiate the presented findings and allow for further subgroup analysis.

## Supplementary Information

Additional file 1. Regression Analysis for the Covariate 'Intake of Antihypertensive Medication.

Additional file 2. Study outcomes.

## Data Availability

No datasets were generated or analysed during the current study.

## Abbreviations

BP: Blood pressure
CDR: Cardiovascular dysregulation
EDA: Electrodermal activity
HR: Heart rate
MTI: Multimodal therapy intervention
PD: Parkinson’s Disease
RMSSD: Root mean sum of squared distance
