# Supplementary material for: Sex-differences in autonomic and cardiovascular responses to multimodal therapy in Parkinson’s disease: a pilot study
Source: BMC Neurol. 2025 Jun 26;25:253. doi: 10.1186/s12883-025-04281-7 (PMC12199527; doi:10.1186/s12883-025-04281-7)
Supplement: Supplementary file 1 — Additional file 1. Regression Analysis for the Covariate 'Intake of Antihypertensive Medication. [file 12883_2025_4281_MOESM1_ESM.pdf]

Supplemental Material:
Regression Analysis for the Covariate *Intake of Antihypertensive Medication*

			**Systolic blood pressure**	**Heart rate**	**RMSSD**	**meanEDA**
**Supine baseline before orthostasis**	males	B	-10.7	3.8	-7.1	2.1
		SE	10.8	1.9	4.5	2.5
		Beta	-.32	.43	-.45	.29
		t	-.99	2.0	-1.6	.86
		Sig.	.35	.08	.16	.42
	females	B	-18.0	1.8	4.7	-.40
		SE	13.6	1.5	16.7	1.7
		Beta	-.55	.27	.21	-.10
		t	-1.3	1.2	.28	-.24
		Sig.	.23	.30	.81	.82
**Adaption to supine rest after orthostasis**	males	B	-13.0	1.5	-.93	2.9
		SE	12.6	2.0	6.1	2.6
		Beta	-.375	.18	-.47	.37
		t	-1.05	.75	-1.5	1.1
		Sig.	.32	.51	.19	.30
	females	B	2.3	3.0	.02	-.12
		SE	7.4	2.4	3.9	1.3
		Beta	.16	.56	.003	-.03
		t	.31	1.3	.01	-.09
		Sig.	.77	.29	1.0	.93

Regression Analysis for the Covariate 'Intake of Antihypertensive Drugs'*; Association between the covariate and the study outcomes Blood pressure [mmHg], Heart rate [bpm], RMSSD [ms], and meanEDA [µs] in baseline state before orthostasis and after orthostasis; for men and women. Linear regression models were used to assess whether the covariate was associated with the change (Δ) in outcome parameters from pre- to post-intervention. B: Unstandardized Coefficient; SE: Standard Error; Beta: standardized coefficient; t: t-value; Sig.: p-value. p-values presented in this table are unadjusted. Bonferroni-Holm correction for multiple comparisons was applied post hoc across all covariates and outcome parameters in the study.*
