# Supplementary material for: Sex-differences in autonomic and cardiovascular responses to multimodal therapy in Parkinson’s disease: a pilot study
Source: BMC Neurol. 2025 Jun 26;25:253. doi: 10.1186/s12883-025-04281-7 (PMC12199527; doi:10.1186/s12883-025-04281-7)
Supplement: Supplementary file 2 — Additional file 2. Study outcomes. [file 12883_2025_4281_MOESM2_ESM.pdf]

Supplemental Material: Study Outcomes

				Systolic blood pressure [mmHg]	Heart rate [bpm]	RMSSD [ms]	meanEDA [µs]
Supine baseline before orthostasis	males	pre MTI	n	22	17	17	17
Supine baseline before orthostasis	males	pre MTI	mean ± SD	127.2 ± 12.9	64.1 ± 10.5	25.1 ± 16.2	1.3 ± 3.4
Supine baseline before orthostasis	males	pre MTI	min - max	109 - 152	37.0 – 82.0	6.15 – 57.22	.02 – 14.11
Supine baseline before orthostasis	males	post MTI	n	17	13	12	13
Supine baseline before orthostasis	males	post MTI	mean ± SD	130.3 ± 17.2	63.9 ± 7.6	27.0 ± 15.8	0.5 ± 0.4
Supine baseline before orthostasis	males	post MTI	min - max	112 - 178	46.0 – 74.0	10.55 – 56.14	.04 – 1.25
Supine baseline before orthostasis	males	pre-post-difference	significance	p=.794	p=.345	p=.583	p=.937
Supine baseline before orthostasis	males	pre-post-difference	Cohen's d	.21	-.02	.12	-.03
Supine baseline before orthostasis	females	pre MTI	n	15	11	11	12
Supine baseline before orthostasis	females	pre MTI	mean ± SD	131.1 ± 25.8	71.4 ± 14.0	23.7 ± 21.0	3.8 ± 7.4
Supine baseline before orthostasis	females	pre MTI	min - max	83 - 198	46.0 – 103.0	3.07 – 77.19	.21 – 26.32
Supine baseline before orthostasis	females	post MTI	n	11	11	11	11
Supine baseline before orthostasis	females	post MTI	mean ± SD	123.4 ± 16.2	67.4 ± 17.8	28.8 ± 23.4	1.4 ± 2.5
Supine baseline before orthostasis	females	post MTI	min - max	98 - 143	38.0 – 109.0	3.95 – 88.87	.02 – 8.12
Supine baseline before orthostasis	females	pre-post-difference	significance	p=.650	p=.725	p=.833	p=.260
Supine baseline before orthostasis	females	pre-post-difference	Cohen's d	-.35	-.25	.23	-.43
Adaption to supine rest after orthostasis	males	pre MTI	n	22	14	14	21
Adaption to supine rest after orthostasis	males	pre MTI	mean ± SD	129.0 ± 14.8	63.5 ± 10.8	29.1 ± 15.2	1.7 ± 3.8
Adaption to supine rest after orthostasis	males	pre MTI	min - max	107 - 161	37.0 – 80.0	11.38 – 57.34	.02 – 16.35
Adaption to supine rest after orthostasis	males	post MTI	n	16	11	11	16
Adaption to supine rest after orthostasis	males	post MTI	mean ± SD	134.2 ± 22.6	65.1 ± 6.3	28.6 ± 16.4	0.6 ± 0.5
Adaption to supine rest after orthostasis	males	post MTI	min - max	84-181	56.0 – 73.0	14.32 – 58.04	.02 – 1.75
Adaption to supine rest after orthostasis	males	pre-post-difference	significance	p=.717	p=.588	p=.401	p=.717
Adaption to supine rest after orthostasis	males	pre-post-difference	Cohen's d	.28	.18	-.03	-.38
Adaption to supine rest after orthostasis	females	pre MTI	n	15	10	10	16
Adaption to supine rest after orthostasis	females	pre MTI	mean ± SD	141.1 ± 24.0	73.7 ± 13.5	24.8 ± 19.6	2.8 ± 5.4
Adaption to supine rest after orthostasis	females	pre MTI	min - max	99 - 200	48.0 – 101.0	8.80 – 77.47	.17 – 21.42
Adaption to supine rest after orthostasis	females	post MTI	n	11	11	11	12
Adaption to supine rest after orthostasis	females	post MTI	mean ± SD	130.0 ± 23.3	64.6 ± 7.8	31.2 ± 16.7	1.2 ± 1.9
Adaption to supine rest after orthostasis	females	post MTI	min - max	93 - 174	51.0 – 78.0	14.4 – 70.2	.02 – 6.33
Adaption to supine rest after orthostasis	females	pre-post-difference	significance	**p=.022***	**p=.020***	p=.779	p=.158
Adaption to supine rest after orthostasis	females	pre-post-difference	Cohen's d	.47	-.84	-.35	-.38
Difference between baseline and adaption to rest	males	pre MTI	significance	p=.390	p=.823	p=.167	**p=.003****
Difference between baseline and adaption to rest	males	pre MTI	Cohen's d	.13	-.06	.25	.11
Difference between baseline and adaption to rest	males	post MTI	significance	p=.074	p=.467	p=.093	**p=.028***
Difference between baseline and adaption to rest	males	post MTI	Cohen's d	.20	.17	.10	.22
Difference between baseline and adaption to rest	females	pre MTI	significance	**p=.015***	**p=.034***	p=.059	p=.638
Difference between baseline and adaption to rest	females	pre MTI	Cohen's d	.40	.17	.05	-.16
Difference between baseline and adaption to rest	females	post MTI	significance	**p=.021***	p=1.0	p=.139	p=.285
Difference between baseline and adaption to rest	females	post MTI	Cohen's d	.34	-.20	.12	-.09

Development of study outcomes over time of treatment*; Blood pressure [mmHg], Heart rate [bpm], RMSSD [ms], and meanEDA [µs] in baseline state before orthostasis and during adaption to rest after orthostasis; for men and women; at pre-testing and post-testing; SD: standard deviation; min – max: lowest and highest values; Cohen's d: effect size (<0.2: no effect; 0.2-0.5: small effect; 0.5-0.8: moderate effect; ≥0.8: strong effect); levels of significance were calculated with Wilcoxon test; significant results are highlighted in bold type.*
